# Case Report: *De novo* multiple intracranial aneurysms following extracranial–intracranial bypass and proximal occlusion for a giant serpentine aneurysm

**DOI:** 10.3389/fsurg.2025.1732464

**Published:** 2025-12-12

**Authors:** Hui Xu, Kristy Latour, Bin Xu, Marco Maria Fontanella, Feng Xu

**Affiliations:** 1 Department of Neurosurgery, Nantong Hospital of Traditional Chinese Medicine, Nantong Hospital to Nanjing University of Chinese Medicine, Nantong, China; 2 Neurosurgery Unit, Department of Medical and Surgical Specialties, Radiological Sciences and Public Health, University of Brescia, Brescia, Italy; 3 Department of Neurosurgery, Huashan Hospital, Shanghai Medical College, Fudan University, Shanghai, China; 4 National Center for Neurological Disorders, Shanghai, China; 5 Shanghai Key Laboratory of Brain Function and Restoration and Neural Regeneration, Shanghai, China; 6 Neurosurgical Institute of Fudan University, Shanghai, China; 7 Shanghai Clinical Medical Center of Neurosurgery, Shanghai, China

**Keywords:** *de novo* aneurysm, giant serpentine aneurysm, extracranial-intracranial bypass, case report, middle cerebral artery

## Abstract

**Background:**

Carotid artery occlusion, whether therapeutic, iatrogenic, atherosclerotic, or congenital, induces profound hemodynamic changes in the cerebral circulation. Collateral channels within the circle of Willis compensate to maintain cerebral perfusion; however, the resulting increases in flow velocity, wall shear stress, and pressure gradients are believed to ultimately contribute to aneurysm formation. While *de novo* aneurysms after hunterian ligation have been described, they typically occur years after treatment. The early occurrence of multiple aneurysms following parent artery occlusion combined with extracranial-intracranial (EC-IC) bypass has seldom been reported. Here, we report a unique case of multiple, non-anastomotic MCA aneurysms developing early after double-barrel bypass and proximal clipping.

**Case description:**

A 27-year-old male with severe headache was found to have a partially thrombosed giant serpentine aneurysm (GSA) of the left M2 inferior trunk. The patient underwent double-barrel superficial temporal artery–middle cerebral artery (STA–MCA) bypass followed by proximal clipping. Postoperative imaging confirmed complete exclusion of the aneurysm and excellent bypass patency. Three months later, acute aphasia and right hemiparesis developed. Angiography revealed two newly formed aneurysms along the left MCA bifurcation point and the M2 superior trunk, which were successfully treated with endovascular coil embolization and parent artery occlusion. At nine-month follow-up, the patient remained neurologically intact with patent bypass grafts and no aneurysm recurrence.

**Conclusions:**

This case illustrates an unusual pattern of early, multiple, non-anastomotic *de novo* MCA aneurysms developing after double-barrel STA–MCA bypass and proximal clipping. The findings highlight how revascularization can alter local pressure gradients and redistribute flow in ways that relieve hemodynamic stress on classic collateral pathways yet create new regions of focal wall shear stress along the ipsilateral MCA.

## Introduction

*De novo* aneurysms were first described by Graf and Hamby in 1964 to define aneurysms developing secondarily to preexisting lesions although anatomically unrelated to the original site (1). Hemodynamic stress has long been recognized to contribute significantly to the pathogenesis of aneurysm formation and growth. Consequently, carotid artery occlusion, whether iatrogenic, atherosclerotic, or congenital has been shown to bring profound alterations in cerebral hemodynamics, leading to compensatory flow through collateral pathways in the circle of Willis to preserve cerebral perfusion.

Charbel et al. used quantitative MR angiography (QMRA) to characterize these hemodynamic changes in patients with unilateral internal carotid artery (ICA) stenosis or occlusion ≥90% (2). Their study demonstrated significantly elevated flow velocity and wall shear stress across the anterior communicating artery (AComA) in patients with ICA occlusion who subsequently developed aneurysms (2). Indeed, *de novo* or growing aneurysms were often located on collateral vessels, namely the AComA and P1 segment, where hemodynamic demands increased following ICA occlusion. Although the majority of reported *de novo* aneurysms occur in the anterior circulation, posterior cerebral artery aneurysms have also been described, particularly after bilateral carotid occlusion (3).

*De novo* aneurysm formation has occasionally been reported also in the post-operative period following Hunterian ligation for complex or giant aneurysms (4, 5). Most of these cases involve the anterior circulation, especially the AComA, and typically arise several years after endovascular carotid occlusion or aneurysm trapping (3, 6).

In the present study, we present a unique case of multiple, early *de novo* aneurysm formation occurring only three months after proximal parent vessel occlusion and double-barrel superficial temporal artery–middle cerebral artery (STA–MCA) bypass performed for a giant serpentine MCA aneurysm. The hemodynamic consequences of EC–IC bypass following parent vessel occlusion and its potential role in either mitigating or predisposing to *de novo* aneurysm formation remain poorly understood due to the rarity of reported cases. To our knowledge, this represents the first reported case of multiple, non-anastomotic, early *de novo* aneurysm formation in the anterior circulation after STA–MCA bypass and aneurysm proximal clipping.

## Case illustration

**Figure 1:**
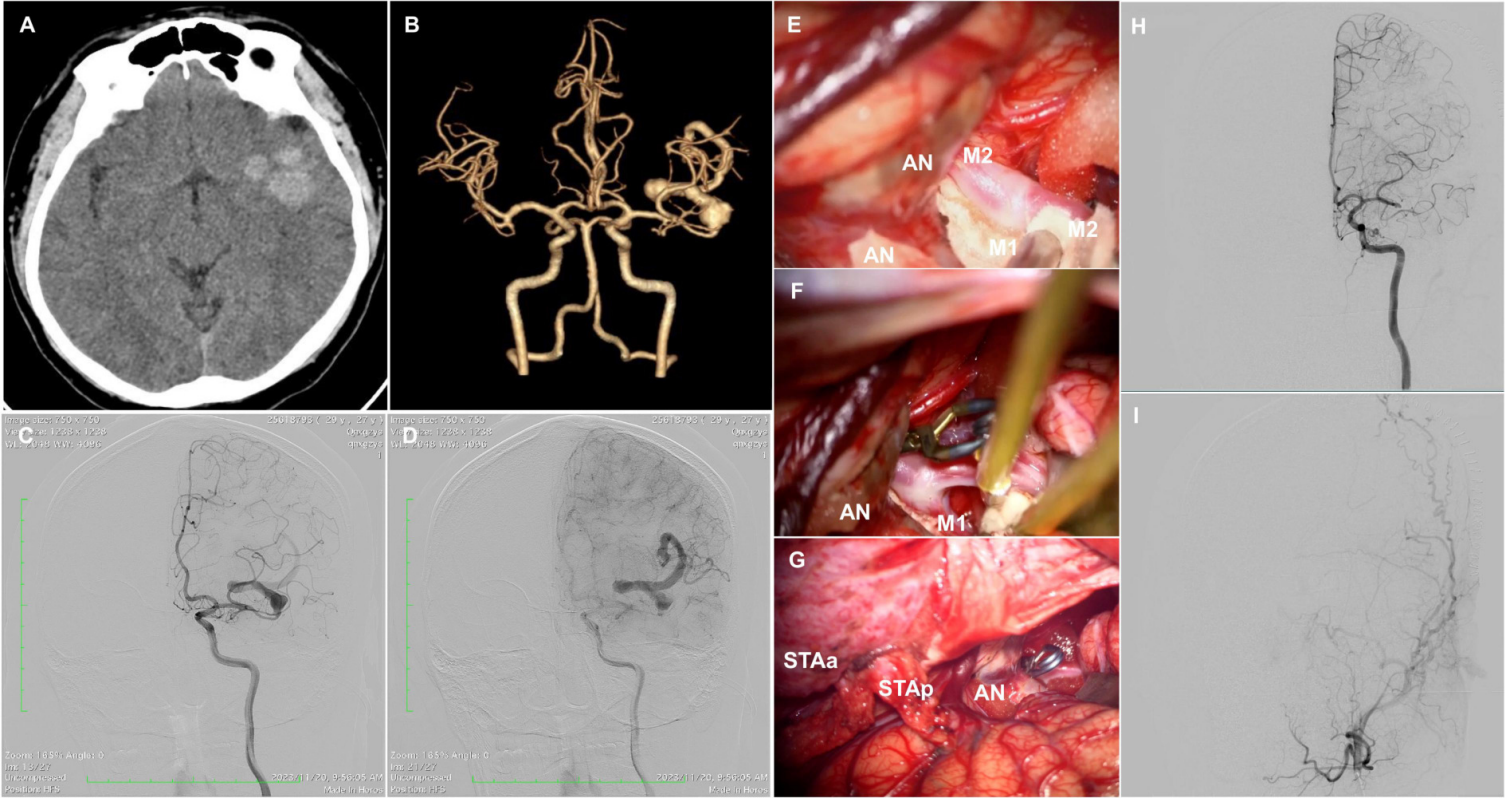
**(A)** Preoperative computed tomography (CT) showing a mixed-density, globoid mass in the left frontotemporal insula. **(B)** CT angiography (CTA) revealing a partially thrombosed giant serpentine aneurysm (GSA) of the left middle cerebral artery (MCA). **(C)** Early-phase digital subtraction angiography (DSA) demonstrating a serpentine vascular channel arising from the inferior trunk of the left MCA. **(D)** Late-phase DSA showing delayed filling of the serpentine channel supplying the distal MCA territory. **(E)** Intraoperative view of the proximal M2 inferior trunk identified proximal to the aneurysm. **(F)** Temporary clipping of the proximal M2 segment performed under neurophysiological monitoring. **(G)** Double-barrel superficial temporal artery–middle cerebral artery (STA–MCA) bypass followed by proximal parent artery occlusion. **(H,I)** Postoperative angiography seven days after surgery demonstrating disappearance of the serpentine lumen and excellent patency of both STA–MCA bypass grafts with distal filling of MCA branches.

In November 2023, a 27-year-old male presented to our institution with a one-month history of severe headache. The patient had no history of smoking, hypertension, or connective tissue disease. On neurological examination, the patient was alert and oriented with intact cranial nerves, full strength (MRC 5/5) in all extremities, normal sensation, and no visual field deficits. Deep tendon reflexes were symmetric, and gait could not be fully assessed due to headache severity but was otherwise unremarkable. Head computed tomography (CT) showed a mixed density globoid mass in the left frontotemporal insula (Figure 1A). CT angiography (CTA) demonstrated a partially thrombosed GSA of the left MCA (Figure 1B). On digital subtraction angiography (DSA), a patent serpentine vascular channel was shown to course through the partially thrombosed aneurysms, with the inflow and outflow of the aneurysmal lumen being distinct and separated by a variable distance. The aneurysm originated from the inferior trunk of the left MCA, and its outflow channel supplied the distal MCA branches and normal cortical territories (Figures 1C,D).

Following discussion of treatment options, microsurgical management was decided, aiming to achieve complete obliteration of the aneurysm, alleviate mass effect, and preserve normal distal vessel perfusion. The surgical strategy was to perform proximal parent artery occlusion combined with distal revascularization through double-barrel STA-MCA bypass. The decision to perform a double-barrel was based on the anticipated high-flow demand of the distal MCA territory following parent artery occlusion. Each bypass was constructed using end-to-side anastomoses with interrupted 10–0 nylon sutures. Following dissection of the frontal and parietal branches of the left STA, a left frontotemporal craniotomy was performed. Wide dissection of the sylvian fissure exposed the M2 inferior trunk just proximal to the aneurysm (Figure 1E), whereas the distal MCA segment exiting the aneurysm could not be exposed. A temporary clip on the proximal M2 inferior segment was applied for 20 min (Figure 1F), during which intraoperative neurophysiological monitoring demonstrated a decrease in both somatosensory evoked potentials (SEP) and motor evoked potentials (MEP). A double-barrel STA-MCA bypass was performed and patency confirmed using intraoperative indocyanine green angiography. The aneurysm was then meticulously dissected, and a permanent clip was applied on its proximal segment (Figure 1G). Intraoperative indocyanine green angiography confirmed immediate graft patency with robust cortical filling. The patient's post-operative course was uneventful. Angiography performed one week after microsurgery demonstrated a marked reduction in aneurysm size, disappearance of the serpentine lumen and complete patency of the double barrel bypass with regular filling of the distal MCA branches (Figures 1H,I). The patient was discharged home neurologically intact on the eighth post-operative day.

**Figure 2:**
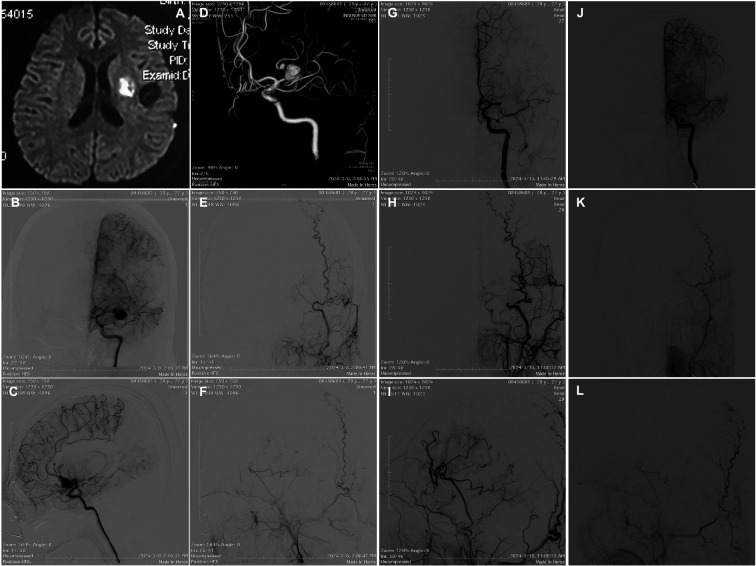
**(A)** Three-months post-operative diffusion-weighted imaging (DWI) showing left periventricular hyperintensity consistent with acute cerebral infarction. **(B,C)** Three-months post-operative DSA revealing two newly developed aneurysms of the left MCA: one located at the bifurcation point and another along the superior M2 trunk. **(D)** Three-months post-operative three-dimensional angiographic reconstruction illustrating the spatial relationship of the aneurysms. **(E,F)** Patency of the double-barrel superficial temporal artery–middle cerebral artery (STA–MCA) bypass was confirmed. **(G)** Transarterial coil embolization of the bifurcation aneurysm and parent artery occlusion performed. **(H,I)** Left external carotid artery injection demonstrating excellent perfusion of the MCA territory through the double-barrel STA–MCA bypass. **(J–L)** Nine-months post-operative gollow-up DSA showing persistent graft patency and complete exclusion of the aneurysms, with no evidence of recurrence.

Three months later, however, the patient was re-admitted to our Institution. At re-presentation, the neurological examination revealed motor aphasia, right facial weakness, and right-sided hemiparesis (MRC 3/5). Diffusion-weighted magnetic resonance imaging (MRI) revealed left periventricular hyperintensity consistent with acute cerebral infarction (Figure 2A). DSA confirmed complete exclusion of the previously treated aneurysm as well as patency of the double-barrel bypass. However, two newly developed aneurysms of the left MCA were identified (Figures 2B–D): one at the bifurcation point and another on the superior M2 trunk.

Given the patency of the bypasses (Figures 2E,F), endovascular management was decided, consisting of transarterial coil embolization of the *de novo* bifurcation aneurysm as well as parent artery occlusion (Figure 2G). The left ICA was catheterized via a 6-French guiding system. A microcatheter was navigated into the newly formed M1-bifurcation aneurysm, which was embolized using detachable platinum coils until complete occlusion was achieved. Parent artery occlusion of the superior M2 branch was then performed using dense coil packing. Balloon- or stent-assisted techniques were avoided due to vessel caliber, acute angulation, and concerns for perforator compromise. No antiplatelet medication was administered before or after the procedure. Post-procedural angiography demonstrated excellent perfusion of the MCA territory via increased flow through the double-barrel STA–MCA (Figures 2H,I). Postoperative course was uneventful, with gradual resolution of aphasia and hemiparesis. The patient was discharged neurologically intact two weeks after treatment. Follow-up DSA at nine months confirmed durable patency of the double-barrel STA–MCA bypass and complete exclusion of the aneurysms (Figures 2J–L).

## Discussion

### Hemodynamic mechanisms in giant serpentine aneurysms

Giant serpentine aneurysms (GSAs) are characterized by a partially thrombosed lumen containing an eccentric, tortuous vascular channel (7).This serpentine flow pattern is thought to arise from the Coandă effect, a hemodynamic phenomenon in which a jet stream is deflected toward one arterial wall rather than coursing centrally, resulting in localized wall stress and progressive aneurysmal remodeling (8).

### *De novo* aneurysm formation after carotid occlusion

Therapeutic carotid artery occlusion, with or without distal revascularization, finds applications in the treatment of complex or giant serpentine aneurysms (9–11). Delayed ischemia and *de novo* aneurysm formation represent however possible long-term sequelae (3, 12). Incidence rates of *de novo* cerebral aneurysms after carotid artery occlusion is a matter of debate and remains yet anecdotal, ranging from 0.7% in certain series to as high as 20% in others, largely reflecting differences in imaging resolution across the last decades (5, 13–15). Earlier reports might not have been adequate to detect previously existing small aneurysms or infundibular dilations. On the other hand, the presence of subarachnoid hemorrhage and potential resultant vasospasm, as well as transient spontaneous thrombosis may mask small lesions. Furthermore, the time interval between parent artery occlusion and *de novo* aneurysm detection has ranged from one day to 20 years, with a mean of 7.4 years and a median of 6.5 years (3).

The hemodynamic consequences of carotid occlusion are substantial. Occlusion of the internal carotid artery results in redistribution of flow through collateral channels, most notably the anterior communicating (ACom) and posterior communicating (PCom) arteries, causing increased wall shear stress that predisposes these vessels to aneurysm formation. Although most reports concern ICA occlusion, similar hemodynamic principles may apply when a major MCA trunk is therapeutically occluded.

### The protective role and risks of STA–MCA bypass

In this context, an STA–MCA bypass may potentially confer a protective effect by augmenting flow from the external carotid system, thereby counterbalancing and reducing the need for collateral vessels' recruitment and hemodynamic stress.

In our case, the M2 inferior segment was encased within a thrombosed mass, with distal MCA branches arising from the aneurysm dome. Preservation of distal perfusion was therefore mandatory, and the aneurysm was treated with proximal parent artery occlusion combined with a double-barrel STA–MCA bypass to maintain retrograde flow. Intraoperative electrophysiological monitoring confirmed the necessity of revascularization prior to definitive occlusion. The initial postoperative course was uneventful, and early angiography demonstrated graft patency and complete aneurysm exclusion.

Unexpectedly, multiple, non-anastomotic, early *de novo* aneurysms developed within three months along the MCA: the same vessel receiving the bypass flow.

### Hemodynamic insights and clinical implications from our case

If the *de novo* aneurysms had arisen distal to the M2 segment, their development could be attributed to a straightforward increase in flow demand through the double-barrel bypass, with augmented bypass inflow exceeding the adaptive capacity of small-caliber cortical branches and thereby generating focal wall stress. This mechanism would parallel that observed after Hunterian ligation without bypass, in which aneurysms tend to form along the ACom or PCom arteries in response to increased collateral demand from the contralateral or posterior circulation (2).

In our case, however, both *de novo* aneurysms occurred proximal to the clip: on the M1-bifurcation segment and on the superior M2 trunk: an anatomical pattern incompatible with a simple high-flow response in the distal MCA territory. Instead, this configuration suggests that flow redistribution and competing inflow vectors are more relevant than absolute flow increase. After proximal occlusion of the inferior M2 trunk, distal MCA territories were supplied retrogradely through the STA–MCA double-barrel bypass, while antegrade flow from the ICA continued to perfuse the proximal MCA. The convergence of these opposing flow directions at the M1–M2 bifurcation likely created regions of oscillatory shear stress, locally elevated pressure gradients, and complex vortex formation: conditions known to promote endothelial dysfunction and aneurysm initiation. Thus, while the bypass may reduce hemodynamic stress along classic collateral pathways (ACom and PCom), it may paradoxically shift the burden of abnormal flow to other intracranial segments, particularly the ipsilateral MCA bifurcation. Flow diversion might have been considered a potential alternative if the proximal MCA aneurysms had had a wide neck, fusiform morphology and no concerns regarding perforator compromise (16).

Although *de novo* aneurysm formation following STA–MCA bypass is rare, it has been reported typically at or near the anastomotic site (17). Early *de novo* aneurysm formation is generally attributed to intraoperative factors, including inadequate suturing, preexisting microscopic wall defects, such as focal degradation of the internal elastic lamina, vessel injury from temporary clipping, or excessive dissection. In our case, the M2 inferior segment was minimally manipulated and occluded using a permanent clip. Moreover, although high-resolution imaging was performed preoperatively, a very small, radiographically occult M1 or M2 aneurysm cannot be definitively excluded.

The periventricular infarct observed three months after surgery is most plausibly explained by a hemodynamic or perforator-related mechanism. Although the double-barrel STA–MCA bypass provided robust retrograde cortical perfusion, the deep perforating arteries arising from the proximal MCA may instead be exposed to a more marginal hemodynamic environment following proximal inferior trunk M2 occlusion. In this setting, subtle systemic factors, such as transient hypotension or dehydration, or progressive local vascular remodeling at the M1–M2 junction could have further reduced perfusion pressure to these perforators, ultimately precipitating ischemia. This mechanism aligns with the infarct location, clinical presentation, and timing of the event, which occurred subacutely after a major rearrangement of MCA flow dynamics.

A hemodynamic insufficiency mechanism rather than thromboembolism might thus have led to transient postoperative mismatch in flow recruitment through the new bypass channels. No antiplatelet or anticoagulant therapy was introduced after the initial bypass, in accordance with our institutional practice when no endovascular devices are used, and it is uncertain whether such treatment would have mitigated the risk of a predominantly hemodynamic event. Nonetheless, a contribution from microembolic phenomena cannot be completely excluded and represents a limitation of this report.

### Future directions and flow measurements

Given these considerations, flow measurements during and immediately after bypass surgery may help identify potentially excessive flow that could predispose to arterial wall stress and aneurysm formation. Flow measurements and surveillance are advisable, particularly in young patients or those with evidence of abnormal postoperative hemodynamics.

## Conclusions

The present report describes a unique case of multiple, non-anastomotic, early *de novo* MCA aneurysms occurring only three months after double-barrel STA–MCA bypass and proximal M2 inferior segment clipping. This unusual presentation underscores the dynamic and sometimes unpredictable hemodynamic changes that follow revascularization. By modulating pressure gradients and redistributing flow through the external carotid system, STA–MCA bypass may reduce hemodynamic stress on classic collateral pathways, such as the ACom and PCom arteries. However, our case illustrates that such revascularization may simultaneously create new regions of altered wall shear stress along the ipsilateral MCA, where *de novo* aneurysms can still develop.

This case illustrates that the hemodynamic consequences of parent vessel occlusion combined with double-barrel STA–MCA bypass are highly segment-specific and may lead to unexpected patterns of focal arterial stress, particularly at sites where antegrade and retrograde flows interact. This underscores the need for careful postoperative surveillance of not only distal bypass territories but also proximal segments subject to complex flow redistribution.

Future studies focusing on quantitative hemodynamic assessment, including flow volume, velocity, wall shear stress, and pressure gradients, are warranted to better understand the mechanisms driving *de novo* aneurysm formation after bypass surgery. Such investigations may help identify high-risk patients, particularly younger individuals or those with genetic or connective tissue–related predispositions to vascular wall fragility.

## Data Availability

The original contributions presented in the study are included in the article/Supplementary Material, further inquiries can be directed to the corresponding author/s.
